# Astragalus membranaceus and Panax notoginseng, the Novel Renoprotective Compound, Synergistically Protect against Podocyte Injury in Streptozotocin-Induced Diabetic Rats

**DOI:** 10.1155/2019/1602892

**Published:** 2019-04-16

**Authors:** Ruonan Zhai, Guihua Jian, Teng Chen, Ling Xie, Rui Xue, Chongting Gao, Niansong Wang, Youhua Xu, Dingkun Gui

**Affiliations:** ^1^Department of Nephrology, Shanghai Jiao Tong University Affiliated Sixth People's Hospital, Shanghai 200233, China; ^2^Shanghai University of Traditional Chinese Medicine, Shanghai 201203, China; ^3^Shanghai Ocean University, Shanghai 201306, China; ^4^Faculty of Chinese Medicine, State Key Laboratory of Quality Research in Chinese Medicine, Macau University of Science and Technology, Taipa, Macao 999078, China

## Abstract

This study was aimed at investigating the synergistical protective effects of Astragalus membranaceus (AG) and Panax notoginseng (NG) on podocyte injury in diabetic rats. Diabetes was induced in rats by a single intraperitoneal injection of streptozotocin at 55 mg/kg. Diabetic rats were then orally administrated with losartan, AG, NG, and AG plus NG (2 : 1) for 12 weeks. Albuminuria, biochemical markers, renal histopathology, and podocyte number per glomerulus were measured. Podocyte apoptosis was determined by triple immunofluorescence labeling including TUNEL assay, WT1, and DAPI. Renal expression of nephrin, *α*-dystroglycan, Bax, Bcl-xl, and Nox4 was evaluated by immunohistochemistry, western blot, and RT-PCR. AG plus NG ameliorated albuminuria, renal histopathology, and podocyte foot process effacement to a greater degree than did AG or NG alone. The number of podocytes per glomerulus, as well as renal expression of nephrin, *α*-dystroglycan, and Bcl-xl, was decreased, while podocyte apoptosis, as well as renal expression of Bax and Nox4, was increased in diabetic rats. All of these abnormalities were partially restored by AG plus NG to a greater degree than did AG or NG alone. In conclusion, AG and NG synergistically ameliorated diabetic podocyte injury partly through upregulation of nephrin, *α*-dystroglycan, and Bcl-xl, as well as downregulation of Bax and Nox4. These findings might provide a novel treatment combination for DN.

## 1. Introduction

Diabetic nephrology (DN) is one of the most frequent complications of diabetes, and end-stage renal disease (ESRD) in almost 50% of patients was attributed to diabetes in developed countries [1]. Diabetes has also become the leading cause of chronic kidney disease in urban Chinese patients since 2011 [2]. Current therapeutic approaches for DN have been limited to drugs acting on glycemic and blood pressure control; however, there is no effective treatment to delay or prevent the progression of DN [3, 4]. Thus, there is an urgent need for the development of novel approaches for treatment of DN. Podocytes are highly specialized, terminally differentiated epithelial cells, which are important for maintaining glomerular permselectivity [5]. Recent studies indicated that podocyte detachment promoted kidney disease in type 2 DN [6]. Podocytes may encounter foot process effacement and podocyte loss in diabetic conditions [7]. Podocyte loss is the strongest predictor of progression of DN [8]. Taken together, podocyte injury played a critical role in the initiation and progression of DN [9]. Increasing amount of data suggested a beneficial role of Traditional Chinese Medicine (TCM) in DN because of its multiple actions and integral regulation [10]. Astragalus membranaceus (AG), one of the most commonly used Chinese herbs, has been widely used as an immune stimulant and antioxidant [11]. It has been reported that Astragalus membranaceus injection reduced albuminuria in DN patients [12]. Our previous study indicated that Astragaloside IV, one of the active components of AG, prevented podocyte apoptosis *in vivo* and *in vitro* [13]. Panax notoginseng (NG) has long been prescribed for prevention and treatment of cardiovascular diseases in China and other Asian countries [14]. Our previous study demonstrated that Notoginsenoside R1, one of the active components of NG, ameliorated podocyte adhesion *in vivo* and *in vitro* [15]. However, the combined effects of AG and NG on DN have not been investigated yet. Therefore, the present study was aimed at examining the combined effects of AG and NG on podocyte injury in diabetic rats and then providing a novel treatment combination for DN.

## 2. Materials and Methods

### 2.1. Drugs and Reagents

Losartan was purchased from Merck Sharp & Dohme Limited (Merck Sharp & Dohme, Australia). AG granule was purchased from Sichuan Baili Pharmaceutical Co. Ltd. (Sichuan, China). NG granule was purchased from China Resources Sanjiu Medical & Pharmaceutical Co. Ltd. (Shenzhen, China). Streptozotocin (STZ) was purchased from Sigma-Aldrich Company (Sigma-Aldrich, USA). Mouse monoclonal anti-Wilms tumor (WT1) (ab212951), rabbit monoclonal anti-Bax (ab32503), rabbit monoclonal anti-Bcl-xl (ab32370), rabbit monoclonal anti-nephrin (ab216341), rabbit monoclonal anti-Nox4 (ab133303), goat anti-rabbit IgG (ab6721), and monoclonal anti-*β*-actin antibodies (ab8226) were purchased from Abcam Biotechnology (Abcam, England). A rabbit polyclonal anti-*α*-dystroglycan (abs125549a) antibody was purchased from Absin Biochemical Company (Absin, Shanghai, China). The FastQuant RT Kit (with gDNase) was purchased from Tiangen Biochemical Technology Co. Ltd. (Beijing, China). ChamQ Universal SYBR qPCR Master Mix was purchased from Vazyme Biotech Co. Ltd. (Nanjing, China).

### 2.2. Animal Study

The animal protocols were approved by the Animal Ethics Committee of Shanghai Jiao Tong University Affiliated Sixth People's Hospital (Animal Welfare Ethics acceptance number: DWLL2018-0334 and Animal Experiment Registration number: DWSY2018-014), Shanghai, China. All the procedures were performed in accordance with the *Guide for the Care and Use of Laboratory Animals* published by the National Institutes of Health. Eight-week-old healthy male Sprague-Dawley rats, weighing between 200 and 250 g, were housed at a clean-grade laboratory animal room in the Animal Laboratory Center of Shanghai Sixth People's Hospital. Rats were housed in an air-conditioned room at 23 ± 1°C on a 12 : 12 h light-dark cycle. All animals were given free access to standard rat chow and water. Diabetes was induced by a single intraperitoneal injection of streptozotocin (STZ, 55 mg/kg), freshly dissolved in citrate buffer (0.1 mol/L). Seventy-two hours after injection of STZ, the blood glucose level was measured from the tail vein. Rats with a blood glucose level over 16.7 mmol/L were considered to be diabetic [16] and selected for the subsequent experiments. Diabetic rats were then randomly divided into 5 groups (*n* = 6/each group), which were treated with saline, losartan (0.01 g·kg^−1^·d^−1^), AG (0.8 g·kg^−1^·d^−1^), NG (0.4 g·kg^−1^·d^−1^), and AG (0.8 g·kg^−1^·d^−1^) plus NG (0.4 g·kg^−1^·d^−1^), respectively. Normal Sprague-Dawley rats were also treated with saline. After being treated for 3 and 12 weeks, 24 h urine was collected with individual metabolic cages. At the end of 12 weeks of treatment, all the rats were sacrificed and blood samples and kidneys were harvested.

### 2.3. Urinary Albumin and Biochemical Markers

After being harvested, the urine samples were centrifuged at 2000 rpm for 10 minutes and the supernatants of urine were measured for the albumin creatinine ratio (ACR) by an automatic biochemistry analyzer (Hitachi Model 7600-120E, Japan). Blood samples were taken from the abdominal aorta, and EDTA was used for anticoagulation; after being harvested, blood samples were centrifuged at 2000 rpm for 10 minutes and the supernatants of blood were measured for serum creatinine and alanine aminotransferase (ALT) by an automatic biochemistry analyzer (Hitachi Model 7600-120E, Japan).

### 2.4. Light Microscopy

Hematoxylin and eosin (H&E) staining and periodic acid-Schiff (PAS) staining were performed on paraffin sections. Slides were dried for 30 minutes at 65°C and then were deparaffinized and rehydrated through dimethylbenzene(I), dimethylbenzene(II), 100% ethanol(I), 100% ethanol(II), 95% ethanol, 90% ethanol, 80% ethanol, and deionized water, 10 minutes for each step. The sections were then subjected to H&E staining and PAS staining and observed under a light microscope (Leica, Germany).

### 2.5. Transmission Electron Microscopy

The renal cortices were prefixed with 2% glutaraldehyde for 2 hours at 4°C and washed with phosphate-buffered saline (PBS) for three times and then were postfixed with 1% osmic acid for 2 hours at 4°C. After step-by-step dehydration with ethyl alcohol, renal cortices were embedded in epoxy resin. Ultrathin sections were prepared with an ultramicrotome (LKB Company, Sweden), stained with uranyl acetate and lead citrate, and were then examined with a Philip electron microscope (Philip CM-120, Netherlands). The number of podocyte foot processes per *μ*m of GBM was calculated using a curvimeter. Three glomeruli were randomly selected from each rat, and 10 electron micrographs were taken in each glomerulus.

### 2.6. Western Blot Analysis

The renal cortex was lysed in radioimmunoprecipitation assay (RIPA) lysis buffer with phenylmethanesulfonyl fluoride (PMSF), loading buffer, and phosphatase on ice, and protein concentration was determined by the bicinchoninic acid protein assay kit (Biosharp, China). Proteins from renal cortex lysates were denatured in boiling water for 10 minutes, loaded to 10% SDS-polyacrylamide gel electrophoresis (SDS-PAGE), and transferred onto nitrocellulose membranes. The membranes were blocked for 1 hour with 5% bovine serum albumin (BSA) in Tris-buffer saline containing 0.1% Tween 20 (TBST) at room temperature. The membrane was incubated overnight at 4°C with a primary antibody, then washed with TBST for five times and incubated with horseradish peroxidase-conjugated secondary antibodies (Beyotime, China) at room temperature for 1 hour and developed with an enhanced chemiluminescence agent. The membranes were placed on the ChemiDoc Touch Imaging System (Bio-Rad Laboratories, USA) to image a protein band, and ImageJ (Adobe Corp., USA) was used to determine band intensity. Protein expression was quantified as the ratio of a specific band to *β*-actin.

### 2.7. Immunohistochemistry

Immunohistochemistry was performed on paraffin sections. After being deparaffinized and rehydrated, antigens were retrieved by boiling in citrate buffer. Slides were blocked with 0.3% H_2_O_2_ for 15 minutes and 5% BSA (Meilunbio, China) for 1 hour. The primary antibody was incubated overnight at 4°C. After being washed with TBST for three times, slides were incubated with peroxidase-conjugated secondary antibodies (Beyotime, China) for 1 hour at room temperature. Images were recorded with a microscope (Leica, Germany). Image analysis was performed using the ImageJ software (Adobe Corp., USA). For quantitative determination of podocyte numbers, the WT1-positive cells were counted in three randomly chosen glomeruli. Other protein expressions were quantified as the ratio of the positive area to the control group.

### 2.8. Immunofluorescence

Podocyte apoptosis was determined by triple immunofluorescence labeling including terminal deoxynucleotidyl transferase-mediated dUTP nick-end labeling (TUNEL) assay, WT1, and 4′,6-diamidino-2-phenylindole (DAPI). The apoptotic cells were determined with the In Situ Cell Death Detection Kit (POD) (Roche, Switzerland) on paraffin-embedded kidney sections. The TUNEL assay was performed according to the manufacturer's instructions. The triple-positive cells, WT1 (red), TUNEL (green), and DAPI (blue), were identified as the apoptotic podocytes. The fluorescent images were examined under a fluorescence microscope (Leica, Germany). The sections were evaluated independently by two blinded investigators.

### 2.9. Quantitative Real-Time Reverse Transcription Polymerase Chain Reaction (RT-PCR)

Total RNA was isolated from the kidney with TRIzol (Invitrogen, USA), and reverse transcription was performed to generate a cDNA template. The relative mRNA levels were determined via fluorogenic quantitative PCR, and *β*-actin was served as an internal reference gene. Specific primers for the use of SYBR Green are as follows: nephrin: 5′-AGAGACT GGGAGAAGAAGAG-3′ (forward) and 5′-AGCAAATCGGACGACAAG-3′ (reverse); Nox4: 5′-CAGTCAAACAGATGGGATACAGA-3′ (forward) and 5′-ATAGAACTGGGTC CACAGCAGA-3′ (reverse); and Bax: 5′-GTGGTTGCCCTCTTCTACTTTG-3′ (forward) and 5′-CACAAAGATGGTCACTGTCTGC-3′ (reverse). The PCR parameters were as follows: 95°C for 30 s followed by 40 cycles of denaturation at 95°C for 10 s and annealing at 60°C for 30 s. The relative mRNA levels were normalized to those of *β*-actin.

### 2.10. Statistical Analysis

SPSS 23.0 software was adopted to perform statistical analysis. All data were presented as mean ± standard deviation (SD). One-way analysis of variance (ANOVA) followed by Fisher's least significant difference (LSD) post hoc test was applied for multiple comparisons. *P* value < 0.05 was considered statistically significant. GraphPad Prism v5 was used for the histograms.

## 3. Results

### 3.1. Effects of AG and NG on Physical and Biochemical Parameters in Diabetic Rats

**Figure 1:**
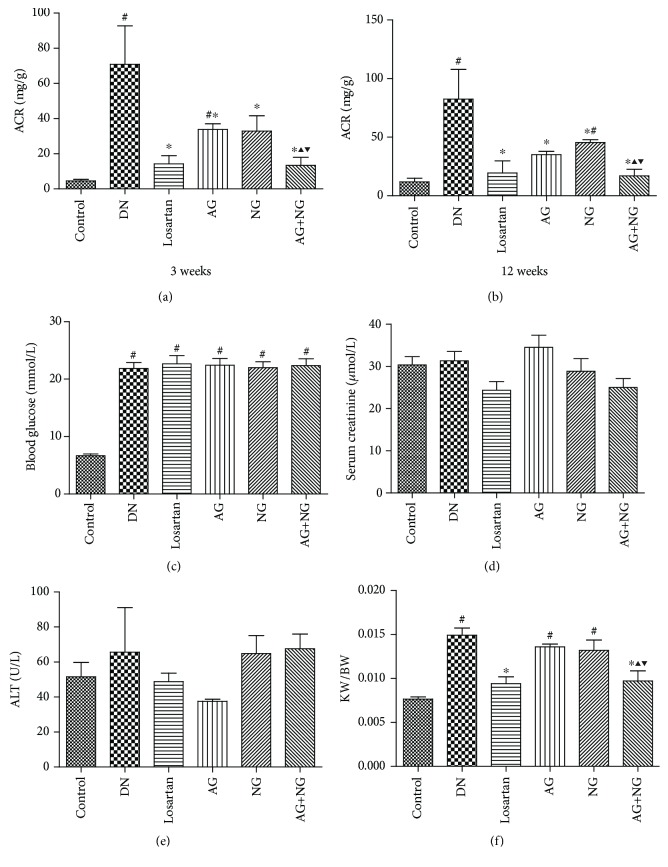
Effects of AG and NG on physical and biochemical parameters in diabetic rats. ACR in diabetic rats after treatment with AG and NG for 3 weeks (a) and 12 weeks (b). Blood glucose (c) after STZ injection. Serum creatinine (d), ALT (e), and KW/BW (f) after treatment with AG and NG for 12 weeks. ACR: albuminuria/creatinine ratio; ALT: alanine aminotransferase; KW/BW: kidney weight per body weight ratio; control: normal control rats; DN: STZ-induced diabetic rats; losartan: DN rats treated with losartan; AG: DN rats treated with Astragalus membranaceus; NG: DN rats treated with Panax notoginseng; AG+NG: DN rats treated with Astragalus membranaceus plus Panax notoginseng. Results were expressed as the mean ± SD (*n* = 6). ^#^*P* < 0.05 vs. control group, ^∗^*P* < 0.05 vs. DN group, ^▲^*P* < 0.05 vs. AG group, ^▼^*P* < 0.05 vs. NG group.

Compared with normal control rats, diabetic rats developed severe albuminuria, at 3 weeks (Figure 1(a)) and 12 weeks (Figure 1(b)) after STZ injection. However, treatment with losartan, AG, NG, and AG plus NG significantly reduced the urinary albumin/creatinine ratio (ACR). Remarkably, AG plus NG reduced ACR to a greater degree than did AG or NG alone at 3 weeks (Figure 1(a)) and 12 weeks (Figure 1(b)) after STZ injection. Blood glucose (Figure 1(c)) was much higher in diabetic rats than in normal control rats. No significant differences in the level of serum creatinine (Figure 1(d)) and ALT (Figure 1(e)) were observed between each group, which indicated that AG, NG, and AG plus NG did not cause apparent toxicity to the liver and kidney. Moreover, diabetic rats showed a higher kidney weight per body weight ratio than normal control rats. However, AG plus NG, rather than AG or NG alone, decreased the kidney weight per body weight ratio in diabetic rats (Figure 1(f)). These results demonstrated that AG and NG synergistically attenuated albuminuria in diabetic rats.

### 3.2. Effects of AG and NG on Renal Histopathology and Podocyte Foot Process Effacement in Diabetic Rats

**Figure 2:**
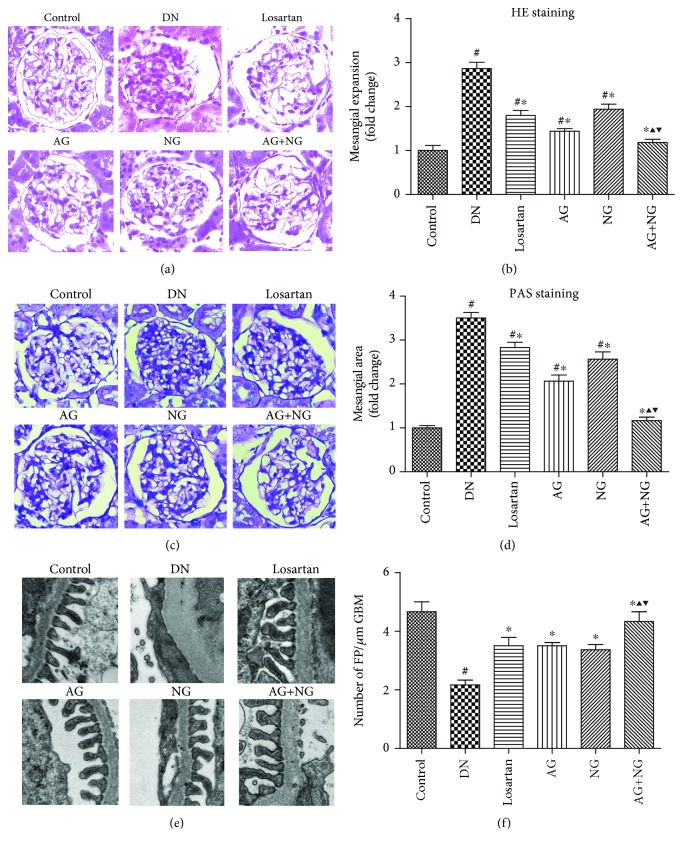
Effects of AG and NG on renal histopathology and podocyte foot process effacement in diabetic rats. Representative hematoxylin and eosin (H&E, kidney histology (×400)) staining (a) and periodic acid-Schiff (PAS, kidney histology (×400)) staining (c) of kidney sections. Representative ultrastructure photos of glomerular podocytes taken by transmission electron microscopy (TEM) (×13500) (e). Semiquantitative analysis of mesangial expansion (b), mesangial area (d), and podocyte foot process density (f). FP: foot process; GBM: glomerular basement membrane; control: normal control rats; DN: STZ-induced diabetic rats; losartan: DN rats treated with losartan; AG: DN rats treated with Astragalus membranaceus; NG: DN rats treated with Panax notoginseng; AG+NG: DN rats treated with Astragalus membranaceus plus Panax notoginseng. Results were expressed as the mean ± SD. ^#^*P* < 0.05 vs. control group, ^∗^*P* < 0.05 vs. DN group, ^▲^*P* < 0.05 vs. AG group, ^▼^*P* < 0.05 vs. NG group.

Twelve weeks after diabetes induction, diabetic rats were characterized with mesangial expansion compared with nondiabetic rats (Figures 2(a) and 2(c)). However, treatment with losartan, AG, NG, and AG plus NG ameliorated mesangial expansion in diabetic rats. Quantitative analysis also revealed a significant improvement in mesangial expansion from the kidneys of rats treated with AG and NG (Figures 2(b) and 2(d)). Transmission electron microscopy images showed apparent podocyte foot process effacement in diabetic rats (Figure 2(e)). However, treatment with losartan, AG, NG, and AG plus NG markedly increased the number of podocyte foot processes per *μ*m of GBM (Figure 2(f)). The effects of AG plus NG on renal histopathology and podocyte foot process effacement were better than those of AG or NG alone. The above results indicated that AG and NG synergistically attenuated renal histopathology and podocyte foot process effacement in diabetic rats.

### 3.3. Effects of AG and NG Treatment on Podocyte Number and mRNA and Protein Levels of Nephrin and *α*-Dystroglycan in STZ-Induced Diabetic Rats

**Figure 3:**
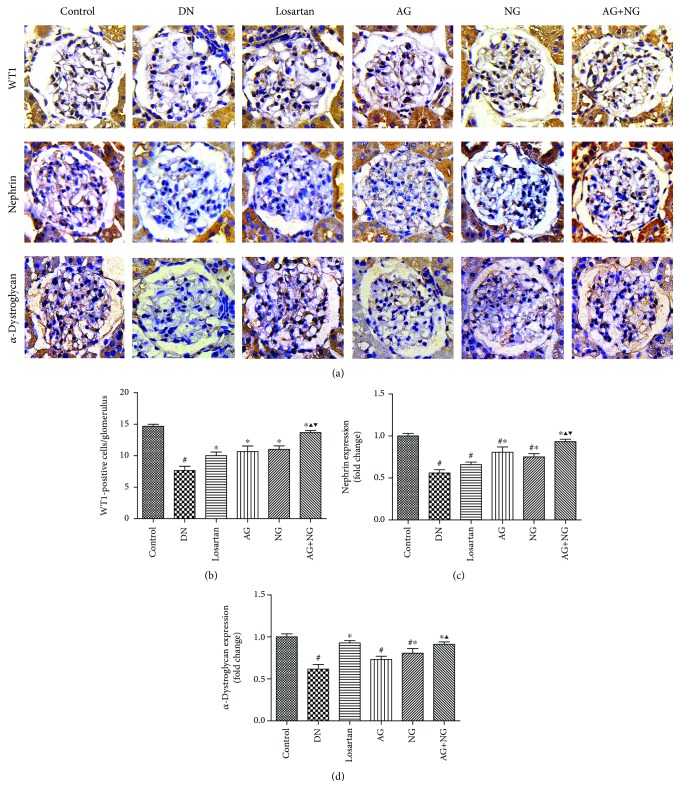
Effects of AG and NG on the expression of WT1, nephrin, and *α*-dystroglycan detected by immunohistochemical staining. Representative photomicrographs of immunostaining for WT1, nephrin, and *α*-dystroglycan (a) in the glomeruli of kidney sections. Quantitative analyses of changes in the number of podocytes per glomerular volume (b). The number of podocytes per glomerulus was determined by counting the number of WT1-expressing nuclei per glomerulus. Semiquantitative analyses of immunostaining for nephrin (c) and *α*-dystroglycan (d) per glomerulus. Control: normal control rats; DN: STZ-induced diabetic rats; losartan: DN rats treated with losartan; AG: DN rats treated with Astragalus membranaceus; NG: DN rats treated with Panax notoginseng; AG+NG: DN rats treated with Astragalus membranaceus plus Panax notoginseng. Results were expressed as the mean ± SD. ^#^*P* < 0.05 vs. control group, ^∗^*P* < 0.05 vs. DN group, ^▲^*P* < 0.05 vs. AG group, ^▼^*P* < 0.05 vs. NG group.

**Figure 4:**
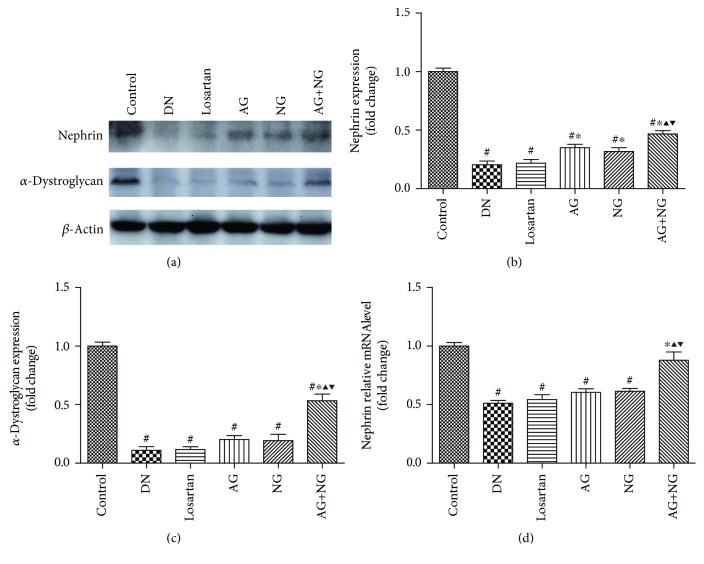
Effects of AG and NG on the levels of nephrin and *α*-dystroglycan detected by western blot and RT-PCR. Representative western blots for nephrin and *α*-dystroglycan (a) in the glomeruli of kidney sections. Semiquantitative analyses of western blot for nephrin (b) and *α*-dystroglycan (c). Glomerular relative mRNA level of nephrin (d). Control: normal control rats; DN: STZ-induced diabetic rats; losartan: DN rats treated with losartan; AG: DN rats treated with Astragalus membranaceus; NG: DN rats treated with Panax notoginseng; AG+NG: DN rats treated with Astragalus membranaceus plus Panax notoginseng. Results were expressed as the mean ± SD. ^#^*P* < 0.05 vs. control group, ^∗^*P* < 0.05 vs. DN group, ^▲^*P* < 0.05 vs. AG group, ^▼^*P* < 0.05 vs. NG group.

To assess podocyte number per glomerulus, the renal tissue sections were immune stained with WT1. The number of podocytes per glomerulus was determined by counting the number of WT1-positive nuclei per glomerulus. At 12 weeks after STZ injection, diabetic rats showed a severe reduction in podocyte number per glomerulus when compared with the normal control rats (Figure 3(a)). However, daily treatment with losartan, AG, NG, and AG plus NG for 12 weeks had a substantial normalizing effect on podocyte density in diabetic rats (Figure 3(b)). Importantly, AG plus NG increased podocyte number per glomerulus to a greater degree than did AG or NG alone. We further investigated the effects of AG and NG on nephrin and *α*-dystroglycan expression in diabetic rats. As shown by immunohistochemical staining (Figure 3(a)) and western blot (Figure 4(a)), the expression of nephrin and *α*-dystroglycan was reduced in the renal tissue from STZ-induced diabetic rats when compared with the normal control rats. However, AG plus NG treatment increased the expression of nephrin and *α*-dystroglycan in diabetic rats. Quantitative analysis also showed a significant increase in the expression of nephrin (Figures 3(c) and 4(b)) and *α*-dystroglycan (Figures 3(d) and 4(c)) from the kidneys of rats treated with AG plus NG. We also studied the effect of AG and NG on the mRNA level of nephrin, and the renal mRNA level of nephrin was reduced in diabetic rats. However, AG plus NG treatment increased the renal mRNA level of nephrin (Figure 4(d)). The above results indicated that AG plus NG synergistically attenuated podocyte loss and restored the mRNA and protein level of nephrin, as well as the expression of *α*-dystroglycan in diabetic rats.

### 3.4. Effects of AG and NG on Podocyte Apoptosis and mRNA and Protein Levels of Bax and Bcl-xl in STZ-Induced Diabetic Rats

**Figure 5:**
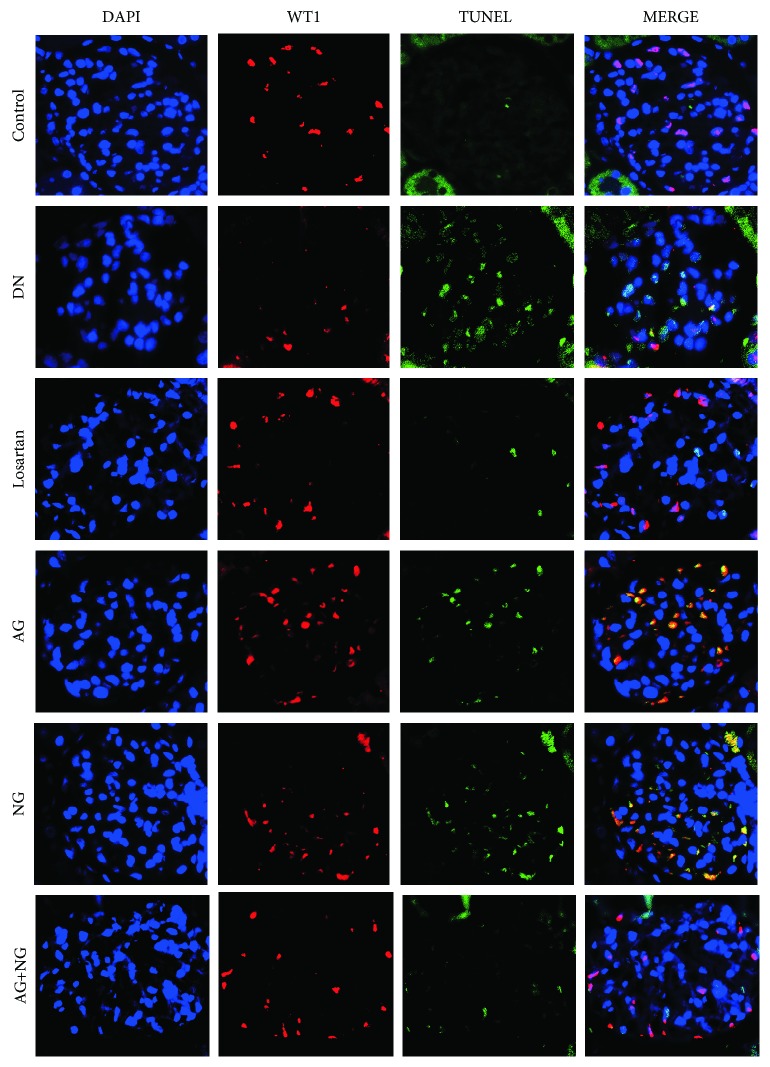
Effects of AG and NG on podocyte apoptosis in diabetic rats. The triple-positive cells, WT1 (red), TUNEL (green), and DAPI (blue), were identified as the apoptotic podocytes. Control: normal control rats; DN: STZ-induced diabetic rats; losartan: DN rats treated with losartan; AG: DN rats treated with Astragalus membranaceus; NG: DN rats treated with Panax notoginseng; AG+NG: DN rats treated with Astragalus membranaceus plus Panax notoginseng.

**Figure 6:**
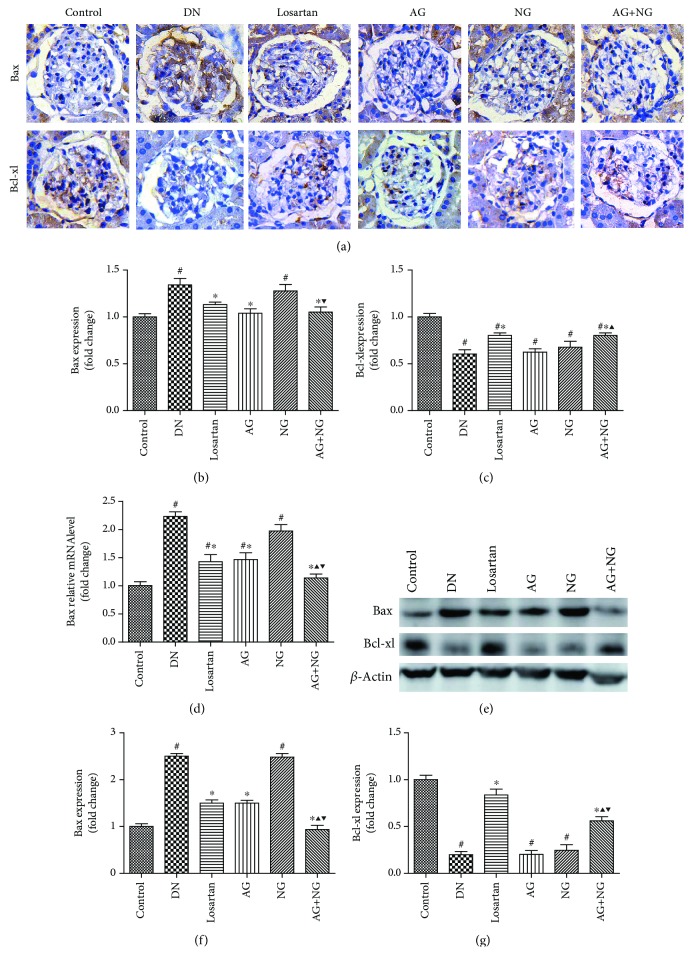
Effects of AG and NG on the expression of Bax and Bcl-xl in diabetic rats. Representative photomicrographs of immunostaining (a) and western blots (e) for Bax and Bcl-xl in the glomeruli of kidney sections. Semiquantitative analyses of immunostaining for Bax (b) and Bcl-xl (c). Glomerular relative mRNA levels of Bax (d). Semiquantitative analyses of western blots for Bax (f) and Bcl-xl (g). Control: normal control rats; DN: STZ-induced diabetic rats; losartan: DN rats treated with losartan; AG: DN rats treated with Astragalus membranaceus; NG: DN rats treated with Panax notoginseng; AG+NG: DN rats treated with Astragalus membranaceus plus Panax notoginseng. Results were expressed as the mean ± SD. ^#^*P* < 0.05 vs. control group, ^∗^*P* < 0.05 vs. DN group, ^▲^*P* < 0.05 vs. AG group, ^▼^*P* < 0.05 vs. NG group.

Podocyte apoptosis was significantly increased in diabetic rats when compared with normal control rats. However, treatment with losartan, AG, NG, and AG plus NG for 12 weeks attenuated podocyte apoptosis in diabetic rats (Figure 5). To reveal the mechanisms underlying the effects of AG plus NG on podocyte apoptosis, the mRNA and protein levels of Bax and Bcl-xl were examined. The Bax expression was elevated, and the Bcl-xl expression was reduced in diabetic rats, as shown by immunohistochemical staining (Figure 6(a)) and western blot (Figure 6(e)). However, treatment with losartan and AG plus NG for 12 weeks restored the Bax and Bcl-xl expression in diabetic rats. Quantitative analysis also showed a significant decrease in Bax expression (Figures 6(b) and 6(f)) and an increase in Bcl-xl expression (Figures 6(c) and 6(g)) from the kidneys of rats treated with AG plus NG. As shown by the RT-PCR study, the mRNA level of Bax was increased in diabetic rats (Figure 6(d)). However, treatment with losartan and AG plus NG for 12 weeks restored the mRNA level of Bax in diabetic rats. The above results demonstrated that AG plus NG synergistically attenuated podocyte apoptosis and restored the balance of Bax and Bcl-xl expression.

### 3.5. Effects of AG and NG on mRNA and Protein Levels of Nox4 in STZ-Induced Diabetic Rats

**Figure 7:**
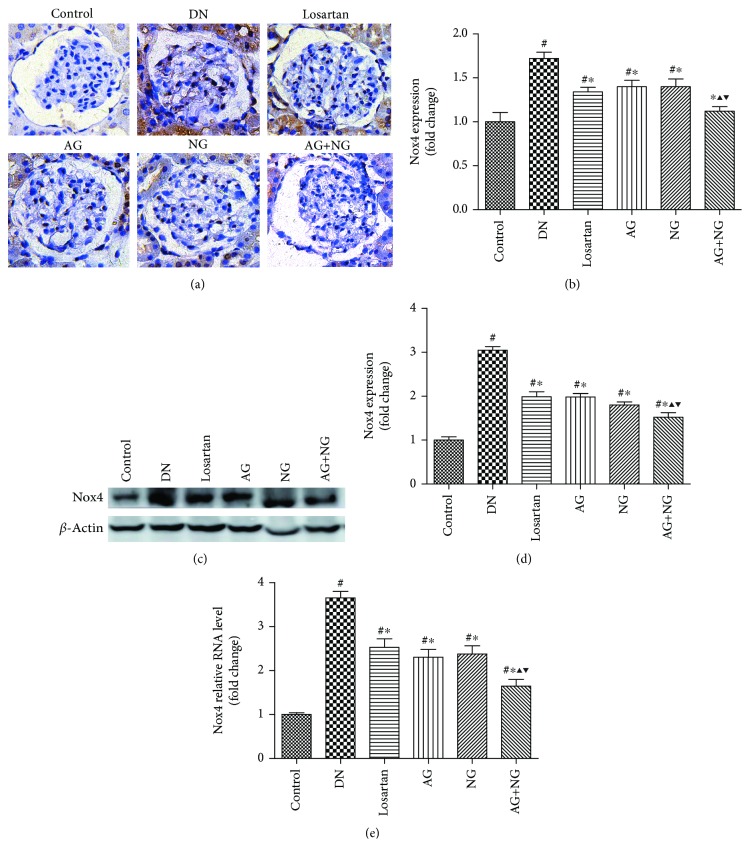
Effects of AG and NG on NADPH oxidase 4 (Nox4) in diabetic rats. Representative photomicrographs of immunostaining (a) and western blots (c) for Nox4 in the glomeruli of kidney sections. Semiquantitative analyses of immunostaining (b) and western blots (d) for Nox4. Glomerular relative mRNA levels of Nox4 (e). Control: normal control rats; DN: STZ-induced diabetic rats; losartan: DN rats treated with losartan; AG: DN rats treated with Astragalus membranaceus; NG: DN rats treated with Panax notoginseng; AG+NG: DN rats treated with Astragalus membranaceus plus Panax notoginseng. Results were expressed as the mean ± SD. ^#^*P* < 0.05 vs. control group, ^∗^*P* < 0.05 vs. DN group, ^▲^*P* < 0.05 vs. AG group, ^▼^*P* < 0.05 vs. NG group.

Compared with the normal control rats, the protein expression and mRNA level of Nox4 were significantly increased in diabetic rats, as shown by immunohistochemical staining (Figure 7(a)), western blot (Figure 7(c)), and RT-PCR (Figure 7(e)). However, treatment with losartan, AG, NG, and AG plus NG for 12 weeks significantly reduced the protein expression and mRNA level of Nox4 in the kidney cortex (Figures 7(b), 7(d), and 7(e)). Remarkably, AG plus NG reduced Nox4 expression and mRNA level to a greater degree than did AG or NG alone. The above results indicated that AG plus NG synergistically reduced Nox4 mRNA and protein levels in diabetic rats.

## 4. Discussion

**Figure 8:**
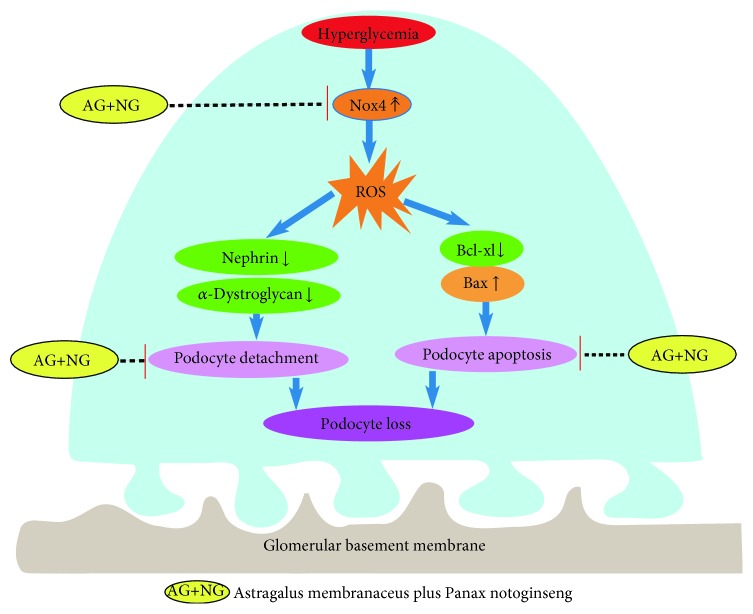
Graphic representation of the potential protection mechanism of AG plus NG on podocyte injury in diabetic rats.

In the present study, we firstly reported that AG plus NG synergistically ameliorated podocyte injury in STZ-induced diabetic rats. Our conclusion was supported by the following findings. (i) AG plus NG attenuated albuminuria, renal histopathology, and podocyte foot process effacement to a greater degree than did AG or NG alone. (ii) AG plus NG increased podocyte number and decreased apoptotic podocytes to a greater degree than did AG or NG alone. (iii) AG plus NG increased renal expression of nephrin, *α*-dystroglycan, and Bcl-xl, while it decreased renal expression of Nox4 to a greater degree than did AG or NG alone. These results clearly demonstrated that the combination of AG and NG synergistically attenuated podocyte injury and subsequent podocyte loss (Figure 8). AG, commonly known as Huangqi, is famous for tonifying qi (Yiqi). NG, commonly known as Sanqi, is famous for invigorating blood (Huoxue). A meta-analysis indicated that the Yiqi Yangyin Huoxue method is beneficial to DN patients in reducing microalbuminuria [17]. In the present study, treatment with losartan, AG, NG, and AG plus NG for 12 weeks significantly reduced albuminuria and improved renal histopathology and podocyte foot process effacement in STZ-induced diabetic rats. The effect of AG or NG on decreasing albuminuria was worse than that of losartan; however, the effect of AG plus NG on decreasing albuminuria was similar to that of losartan. These results clearly demonstrated that AG and NG synergistically attenuated the structural and functional abnormalities in diabetic rats. These findings provide a novel treatment combination for DN and other kidney diseases affecting podocytes.

Podocyte injury plays a critical role in the progression of DN. It has been reported that the number of podocytes is decreased in the glomeruli of diabetic patients [18] and diabetic animal models [19]. The reduction in podocyte density is the strongest predictor of progressive DN [20]. Podocyte depletion may lead to glomerular sclerosis and progressively renal dysfunction [21]. Studies have shown that the number of podocytes is the most powerful predictor of renal prognosis in type 2 DM patients [22]. WT1 is expressed throughout life in the podocyte nucleus and used as a specific marker for podocytes [23, 24]. Thus, we used WT1 immunohistochemistry staining for podocyte nuclei and measured the podocyte number per glomerulus. In this study, diabetic rats showed a significant reduction in podocyte number per glomerulus. However, daily treatment with losartan, AG, NG, and AG plus NG for 12 weeks had a substantial normalizing effect on podocyte density in diabetic rats, and the effect of AG plus NG was better than those of losartan, AG, and NG. These results indicated that AG plus NG ameliorated podocyte loss in diabetic rats. The main cause underlying podocyte loss has been considered to be detachment and apoptosis [25, 26]. Detached viable podocytes have been found in the urine of DN patients [27], suggesting that impaired podocyte adhesion to the glomerular basement membrane (GBM) may be a pivotal step in the development of DN. Nephrin is the central component of podocyte slit diaphragm [28]. Nephrin functions as a signaling scaffold, influencing signal transduction pathways which control podocyte adhesion, shape, and survival [29]. *α*-Dystroglycan is localized to basal cell membrane domains of the podocyte and stabilizes podocytes on the GBM [30]. Splitting of dystroglycan-matrix interaction leads to podocyte flattening and disorder of GBM [31]. In this study, diabetic rats showed reduced expression of nephrin and *α*-dystroglycan in the renal cortex. However, treatment with AG plus NG upregulated the expression of nephrin and *α*-dystroglycan in diabetic rats, and the effects of AG plus NG were better than those of losartan, AG, and NG. These results indicated that AG plus NG ameliorated podocyte detachment partly by increasing the expression of nephrin and *α*-dystroglycan in diabetic rats.

We also investigated the effects of AG plus NG on podocyte apoptosis and the underlying mechanisms. Podocyte apoptosis was accurately detected by triple immunofluorescence labeling including TUNEL assay, WT1, and DAPI. We found that diabetic rats showed more apoptotic podocytes, which was consistent with previous research results [32, 33]. Treatment with losartan, AG, NG, and AG plus NG ameliorated podocyte apoptosis in diabetic rats. We then examined the expression of Bax and Bcl-xl expression in diabetic rats to further explore the antiapoptotic effects of AG plus NG. Apoptosis is triggered by either an intrinsic pathway or an extrinsic pathway; the intrinsic apoptotic pathway is controlled by the family of B-cell lymphoma-2 proteins [34]. Bax is activated by stress stimulus, then accumulates on mitochondria and increases the permeability of the mitochondrial outer membrane, resulting in the release of cytochrome C, which in turn promotes the apoptotic process [35]. Bcl-xl acts as an inhibitor of Bax by preventing Bax accumulation to mitochondria and thus prevents the apoptotic process [36]. In this study, diabetic rats showed increased expression of Bax and reduced expression of Bcl-xl in kidneys. However, treatment with losartan and AG plus NG reduced Bax expression and increased Bcl-xl expression, and the effect of AG plus NG was better than that of losartan on Bax expression and was equal with that of losartan on Bcl-xl expression. These results indicated that AG plus NG inhibited podocyte apoptosis partly through restoring the balance of Bax and Bcl-xl expression in diabetic rats.

Finally, we examined the effects of AG plus NG on oxidative stress in diabetic rats. NADPH oxidases are one of the major causes of reactive oxygen species (ROS), which is a key event influencing the pathogenesis of DN [37]. During the progression of DN, ROS is an important second messenger of apoptosis and inflammation-related signaling pathways [38]. Exposure to ROS leads to deglycosylation of *α*-dystroglycan and subsequent podocyte detachment [39]. Glucose-induced ROS also cause podocyte apoptosis and podocyte depletion at the onset of diabetic nephropathy [20, 40]. It was reported that Astragaloside IV inhibited oxidative stress in renal proximal tubular cells [41] and Panax notoginseng saponins protected the kidney from diabetes by activating antioxidant proteins in rats [42]. Nox4 is one of the isoforms of NADPH oxidases and is highly expressed in renal tissues [43]. Nox4-derived ROS participate in the pathological process of DN by reducing glucose tolerance, increasing the production of proteinuria, and promoting renal fibrosis [44]. Nox4 expression was upregulated in diabetic mice and genetic depletion or pharmacologic inhibition of Nox4 provided renoprotection in long-term DN [45, 46], while specific induction of Nox4 expression in podocytes can lead to typical pathological changes of DN in rats' kidneys [47]. In this study, the kidney of diabetic rats showed elevated expression of Nox4 compared with the kidney of nondiabetic rats. While treatment with losartan, AG, NG, and AG plus NG for 12 weeks significantly reduced Nox4 expression in diabetic rats, the effect of AG plus NG was better than those of losartan, AG, and NG. These results indicated that AG plus NG protected against podocyte injury partly by inhibiting oxidative stress in diabetic rats.

We also examined the serum creatinine and ALT levels and blood glucose in rats, and AG plus NG exhibited no effects on the levels of serum creatinine and ALT, indicating that AG plus NG did not cause apparent toxicity to the kidney and liver. Diabetic rats were randomly divided into four groups, namely, diabetic rats (DN) and diabetic rats treated with AG, NG, and AG plus NG. Figure 1(c) shows the level of blood glucose at baseline and at the period when AG, NG, and AG plus NG treatment was not started. Therefore, there was no significant difference in the level of blood glucose among these three groups.

According to the package inserts, the doses of AG and NG in human being was 8 g·d^−1^ [48] and 4 g·d^−1^ [49], respectively. According to the FDA guidance [50], the doses of AG and NG in rats were 0.76 g·kg^−1^·d^−1^ and 0.38 g·kg^−1^·d^−1^, respectively, equivalent to the doses used in human, so the doses we adopted in this study were 0.8 g·kg^−1^·d^−1^ and 0.4 g·kg^−1^·d^−1^, respectively.

There are limitations in this study. First, we only performed *in vivo* studies and the effects of AG and NG on podocytes cultured in high glucose condition need to be further investigated. Second, we cannot be sure that the results from the rodent model can be translated to human. Therefore, the effects of AG and NG in a clinical setting need to be further investigated.

## 5. Conclusions

Taken together, AG and NG, the novel renoprotective compound, synergistically ameliorated diabetic podocyte injury partly through upregulation of nephrin, *α*-dystroglycan, and Bcl-xl, as well as downregulation of Bax and inhibition of oxidative stress. These findings might provide a novel treatment combination for DN.

## Data Availability

The data that support the findings of this study are available from the corresponding authors upon reasonable request.
